# Supplementary material for: Fusion Tag Design Influences Soluble Recombinant Protein Production in Escherichia coli
Source: Int J Mol Sci. 2022 Jul 12;23(14):7678. doi: 10.3390/ijms23147678 (PMC9321918; doi:10.3390/ijms23147678)
Supplement: Supplementary file 1 [file ijms-23-07678-s001.zip › ijms-1778311-supplementary.pdf]

CASPON tag design

Table S1. Amino acid and nucleotide sequences of all tag elements; When used as first element of the fusion tag, the start codon ATG coding for Methionine was added to the respective tag element.

Tag element	Amino acid sequence	Nucleotide sequence
T7AC	LEDPERNKERKEAELQA QTAEQ	CTGGAGGATCCGGAACGCAACAAAGAGCGAAAGGA AGCTGAGTTGCAAGCTCAAACCGCTGAGCAA
T7A3	LEDPERNKERKEAELEA ETAEQ	CTGGAGGATCCGGAACGCAACAAAGAGCGAAAGGA AGCTGAGTTGGAAGCTGAGACCGCTGAGCAA
6-His	HHHHHH	CACCATCATCACCATCAT
L_1	GSG	GGCAGCGGC
L_2	SA	AGCGCG
StrepII	WSHPQFEK	TGGAGCCATCCGCAGTTTGAAAAA
CS	VDVAD	GTGGATGTGGCGGAT

Nucleotide and amino acid sequences of model proteins

Human Fibroblast Growth Factor-2 variants

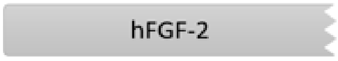

Nucleotide sequence:
ATGGCCGCTGGTTCGATTACTACCCTGCCTGCTTTACCTGAAGATGGTGGTTCTGGTGCGTTCCCGCCGGGTCA
CTTCAAAGACCCAAAACGTTTGTACTGTAAAAACGGTGGCTTTTTTCTGCGCATCCATCCGGACGGCCGCGTGG
ATGGTGTCCGTGAAAAGTCCGACCCGCACATTAAGCTGCAACTGCAGGCCGAGGAGCGTGGTGTTGTTAGCAT
CAAAGGCGTGAGCGCAAATCGTTACCTGGCGATGAAAGAGGATGGCCGTCTGCTGGCGAGCAAGAGCGTTAC
CGACGAGTGCTTCTTCTTTGAACGCCTGGAGAGCAATAATTACAACACCTACCGTAGCCGCAAGTATACCTCTT
GGTATGTGGCGCTGAAGCGTACGGGCCAGTATAAATTGGGTAGCAAAACGGGTCCGGGCCAAAAGGCAATCC
TGTTCCTGCCGATGAGCGCGAAATCC

Amino acid sequence:
MAAGSITTLPALPEDGGSGAFPPGHFKDPKRLYCKNGGFFLRIHPDGRVDGVREKSDPHIKLQLQAEERGVVSIKGV
SANRYLAMKEDGRLLASKSVTDECFFFERLESNNYNTYRSRKYTSWYVALKRTGQYKLGSKTGPGQKAILFLPMSAK
S

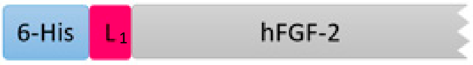

Nucleotide sequence:
ATGCACCATCATCACCATCATGGCAGCGGCGCCGCTGGTTCGATTACTACCCTGCCTGCTTTACCTGAAGATGG
TGGTTCTGGTGCGTTCCCGCCGGGTCACTTCAAAGACCCAAAACGTTTGTACTGTAAAAACGGTGGCTTTTTC
TGCGCATCCATCCGGACGGCCGCGTGGATGGTGTCCGTGAAAAGTCCGACCCGCACATTAAGCTGCAACTGCA
GGCCGAGGAGCGTGGTGTTGTTAGCATCAAAGGCGTGAGCGCAAATCGTTACCTGGCGATGAAAGAGGATG
GCCGTCTGCTGGCGAGCAAGAGCGTTACCGACGAGTGCTTCTTCTTTGAACGCCTGGAGAGCAATAATTACAA
CACCTACCGTAGCCGCAAGTATACCTCTTGGTATGTGGCGCTGAAGCGTACGGGCCAGTATAAATTGGGTAGC
AAAACGGGTCCGGGCCAAAAGGCAATCCTGTTCCTGCCGATGAGCGCGAAATCC

Amino acid sequence:
MHHHHHHGSGAAGSITTLPALPEDGGSGAFPPGHFKDPKRLYCKNGGFFLRIHPDGRVDGVREKSDPHIKLQLQA
EERGVVSIKGVSANRYLAMKEDGRLLASKSVTDECFFFERLESNNYNTYRSRKYTSWYVALKRTGQYKLGSKTGPGQ
KAILFLPMSAKS

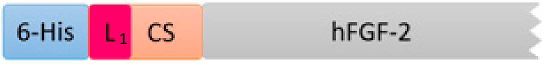

Nucleotide sequence:
ATGCACCATCATCACCATCATGGCAGCGGCGTGGATGTGGCGGATGCCGCTGGTTCGATTACTACCCTGCCTG
CTTTACCTGAAGATGGTGGTTCTGGTGCGTTCCCGCCGGGTCACTTCAAAGACCCAAAACGTTTGTACTGTAAA
AACGGTGGCTTTTTTCTGCGCATCCATCCGGACGGCCGCGTGGATGGTGTCCGTGAAAAGTCCGACCCGCACA
TTAAGCTGCAACTGCAGGCCGAGGAGCGTGGTGTTGTTAGCATCAAAGGCGTGAGCGCAAATCGTTACCTGG
CGATGAAAGAGGATGGCCGTCTGCTGGCGAGCAAGAGCGTTACCGACGAGTGCTTCTTCTTTGAACGCCTGG
AGAGCAATAATTACAACACCTACCGTAGCCGCAAGTATACCTCTTGGTATGTGGCGCTGAAGCGTACGGGCCA
GTATAAATTGGGTAGCAAAACGGGTCCGGGCCAAAAGGCAATCCTGTTCCTGCCGATGAGCGCGAAATCC

Amino acid sequence:
MHHHHHHGSGVDVADAAGSITTLPALPEDGGSGAFPPGHFKDPKRLYCKNGGFFLRIHPDGRVDGVREKSDPHIK
LQLQAEERGVVSIKGVSANRYLAMKEDGRLLASKSVTDECFFFERLESNNYNTYRSRKYTSWYVALKRTGQYKLGSK
TGPGQKAILFLPMSAKS

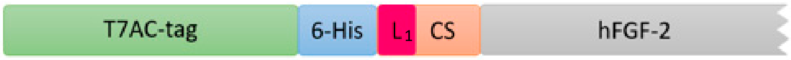

Nucleotide sequence:
ATGCTGGAGGATCCGGAACGCAACAAAGAGCGAAAGGAAGCTGAGTTGCAAGCTCAAACCGCTGAGCAACA
CCATCATCACCATCATGGCAGCGGCGTGGATGTGGCGGATGCCGCTGGTTCGATTACTACCCTGCCTGCTTTAC
CTGAAGATGGTGGTTCTGGTGCGTTCCCGCCGGGTCACTTCAAAGACCCAAAACGTTTGTACTGTAAAAACGG
TGGCTTTTTTCTGCGCATCCATCCGGACGGCCGCGTGGATGGTGTCCGTGAAAAGTCCGACCCGCACATTAAG
CTGCAACTGCAGGCCGAGGAGCGTGGTGTTGTTAGCATCAAAGGCGTGAGCGCAAATCGTTACCTGGCGATG
AAAGAGGATGGCCGTCTGCTGGCGAGCAAGAGCGTTACCGACGAGTGCTTCTTCTTTGAACGCCTGGAGAGC

AATAATTACAACACCTACCGTAGCCGCAAGTATACCTCTTGGTATGTGGCGCTGAAGCGTACGGGCCAGTATA
AATTGGGTAGCAAAACGGGTCCGGGCCAAAAGGCAATCCTGTTCCTGCCGATGAGCGCGAAATCC

Amino acid sequence:
MLEDPERNKERKEAELQAQTAEQHHHHHHGSGVDVADAAGSITTLPALPEDGGSGAFPPGHFKDPKRLYCKNGG
FFLRIHPDGRVDGVREKSDPHIKLQLQAEERGVVSIKGVSANRYLAMKEDGRLLASKSVTDECFFFERLESNNYNTYR
SRKYTSWYVALKRTGQYKLGSKTGPGQKAILFLPMSAKS

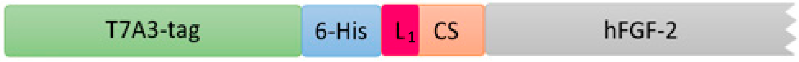

Nucleotide sequence:
ATGCTGGAGGATCCGGAACGCAACAAAGAGCGAAAGGAAGCTGAGTTGGAAGCTGAGACCGCTGAGCAACA
CCATCATCACCATCATGGCAGCGGCGTGGATGTGGCGGATGCCGCTGGTTCGATTACTACCCTGCCTGCTTTAC
CTGAAGATGGTGGTTCTGGTGCGTTCCCGCCGGGTCACTTCAAAGACCCAAAACGTTTGTACTGTAAAAACGG
TGGCTTTTTTCTGCGCATCCATCCGGACGGCCGCGTGGATGGTGTCCGTGAAAAGTCCGACCCGCACATTAAG
CTGCAACTGCAGGCCGAGGAGCGTGGTGTTGTTAGCATCAAAGGCGTGAGCGCAAATCGTTACCTGGCGATG
AAAGAGGATGGCCGTCTGCTGGCGAGCAAGAGCGTTACCGACGAGTGCTTCTTCTTTGAACGCCTGGAGAGC
AATAATTACAACACCTACCGTAGCCGCAAGTATACCTCTTGGTATGTGGCGCTGAAGCGTACGGGCCAGTATA
AATTGGGTAGCAAAACGGGTCCGGGCCAAAAGGCAATCCTGTTCCTGCCGATGAGCGCGAAATCC

Amino acid sequence:
MLEDPERNKERKEAELEAETAEQHHHHHHGSGVDVADAAGSITTLPALPEDGGSGAFPPGHFKDPKRLYCKNGGF
FLRIHPDGRVDGVREKSDPHIKLQLQAEERGVVSIKGVSANRYLAMKEDGRLLASKSVTDECFFFERLESNNYNTYRS
RKYTSWYVALKRTGQYKLGSKTGPGQKAILFLPMSAKS

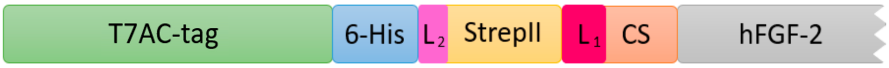

Nucleotide sequence:
ATGCTGGAGGATCCGGAACGCAACAAAGAGCGAAAGGAAGCTGAGTTGCAAGCTCAAACCGCTGAGCAACA
CCATCATCACCATCATAGCGCGTGGAGCCATCCGCAGTTTGAAAAAGGCAGCGGCGTGGATGTGGCGGATGC
CGCTGGTTCGATTACTACCCTGCCTGCTTTACCTGAAGATGGTGGTTCTGGTGCGTTCCCGCCGGGTCACTTCA
AAGACCCAAAACGTTTGTACTGTAAAAACGGTGGCTTTTTTCTGCGCATCCATCCGGACGGCCGCGTGGATGG
TGTCCGTGAAAAGTCCGACCCGCACATTAAGCTGCAACTGCAGGCCGAGGAGCGTGGTGTTGTTAGCATCAAA
GGCGTGAGCGCAAATCGTTACCTGGCGATGAAAGAGGATGGCCGTCTGCTGGCGAGCAAGAGCGTTACCGAC
GAGTGCTTCTTCTTTGAACGCCTGGAGAGCAATAATTACAACACCTACCGTAGCCGCAAGTATACCTCTTGGTA
TGTGGCGCTGAAGCGTACGGGCCAGTATAAATTGGGTAGCAAAACGGGTCCGGGCCAAAAGGCAATCCTGTT
CCTGCCGATGAGCGCGAAATCCTAA

Amino acid sequence:
MLEDPERNKERKEAELQAQTAEQHHHHHHSAWSHPQFEKGSGVDVADAAGSITTLPALPEDGGSGAFPPGHFKD
PKRLYCKNGGFFLRIHPDGRVDGVREKSDPHIKLQLQAEERGVVSIKGVSANRYLAMKEDGRLLASKSVTDECFFFER
LESNNYNTYRSRKYTSWYVALKRTGQYKLGSKTGPGQKAILFLPMSAKS

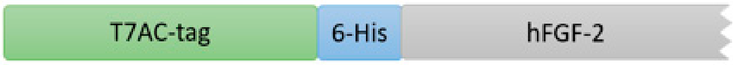

Nucleotide sequence:
ATGCTGGAGGATCCGGAACGCAACAAAGAGCGAAAGGAAGCTGAGTTGCAAGCTCAAACCGCTGAGCAACA
CCATCATCACCATCATGCCGCTGGTTCGATTACTACCCTGCCTGCTTTACCTGAAGATGGTGGTTCTGGTGCGTT

CCCGCCGGGTCACTTCAAAGACCCAAAACGTTTGTACTGTAAAAACGGTGGCTTTTTCTGCGCATCCATCCGG
ACGGCCGCGTGGATGGTGTCCGTGAAAAGTCCGACCCGCACATTAAGCTGCAACTGCAGGCCGAGGAGCGTG
GTGTTGTTAGCATCAAAGGCGTGAGCGCAAATCGTTACCTGGCGATGAAAGAGGATGGCCGTCTGCTGGCGA
GCAAGAGCGTTACCGACGAGTGCTTCTTCTTTGAACGCCTGGAGAGCAATAATTACAACACCTACCGTAGCCG
CAAGTATACCTCTTGGTATGTGGCGCTGAAGCGTACGGGCCAGTATAAATTGGGTAGCAAAACGGGTCCGGG
CCAAAAGGCAATCCTGTTCCTGCCGATGAGCGCGAAATCC

Amino acid sequence:
MLEDPERNKERKEAELQAQTAEQHHHHHHAAGSITTLPALPEDGGSGAFPPGHFKDPKRLYCKNGGFFLRIHPDG
RVDGVREKSDPHIKLQLQAEERGVVSIKGVSANRYLAMKEDGRLLASKSVTDECFFFERLESNNYNTYRSRKYTSWY
VALKRTGQYKLGSKTGPGQKAILFLPMSAKS

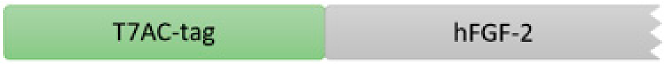

Nucleotide sequence:
ATGCTGGAGGATCCGGAACGCAACAAAGAGCGAAAGGAAGCTGAGTTGCAAGCTCAAACCGCTGAGCAAGC
CGCTGGTTCGATTACTACCCTGCCTGCTTTACCTGAAGATGGTGGTTCTGGTGCGTTCCCGCCGGGTCACTTCA
AAGACCCAAAACGTTTGTACTGTAAAAACGGTGGCTTTTTCTGCGCATCCATCCGGACGGCCGCGTGGATGG
TGTCCGTGAAAAGTCCGACCCGCACATTAAGCTGCAACTGCAGGCCGAGGAGCGTGGTGTTGTTAGCATCAAA
GGCGTGAGCGCAAATCGTTACCTGGCGATGAAAGAGGATGGCCGTCTGCTGGCGAGCAAGAGCGTTACCGAC
GAGTGCTTCTTCTTTGAACGCCTGGAGAGCAATAATTACAACACCTACCGTAGCCGCAAGTATACCTCTTGGTA
TGTGGCGCTGAAGCGTACGGGCCAGTATAAATTGGGTAGCAAAACGGGTCCGGGCCAAAAGGCAATCCTGTT
CCTGCCGATGAGCGCGAAATCC

Amino acid sequence:
MLEDPERNKERKEAELQAQTAEQAAGSITTLPALPEDGGSGAFPPGHFKDPKRLYCKNGGFFLRIHPDGRVDGVRE
KSDPHIKLQLQAEERGVVSIKGVSANRYLAMKEDGRLLASKSVTDECFFFERLESNNYNTYRSRKYTSWYVALKRTG
QYKLGSKTGPGQKAILFLPMSAKS

Mature Tumour Necrosis Factor α

ompA_6-His_L_1_CS_mTNFα

Nucleotide sequence:
ATGAAAAAGACAGCTATCGCGATTGCAGTGGCACTGGCTGGTTTCGCTACCGTAGCGCAGGCCCACCATCATC
ACCATCATGGCAGCGGCGTGGATGTGGCGGATGTCCGTTCCAGCAGCCGTACGCCGAGCGATAAACCTGTCG
CGCACGTAGTGGCGAATCCGCAAGCCGAGGGTCAGCTGCAGTGGCTGAATCGTCGCGCGAACGCGCTGCTGG
CCAATGGTGTTGAGCTGCGTGACAACCAACTGGTTGTTCCATCCGAAGGCCTGTACCTGATTTATTCTCAAGTG
CTGTTCAAAGGTCAGGGTTGCCCGAGCACGCACGTGTTGCTGACCCATACCATTAGCCGCATCGCAGTCAGCT
ACCAGACCAAGGTCAACCTGTTGAGCGCGATCAAGTCCCCGTGTCAACGTGAAACGCCTGAGGGCGCTGAGG
CCAAGCCGTGGTATGAGCCGATCTACCTGGGTGGCGTGTTTCAGCTGGAGAAAGGTGACCGTCTGAGCGCGG
AAATCAACCGCCCGGATTATTTAGATTTTGCCGAGTCTGGTCAGGTGTACTTCGGCATTATTGCACTG

Amino acid sequence:
MKKTAIAIAVALAGFATVAQAHHHHHHGSGVDVADVRSSSRTPSDKPVAHVVANPQAEGQLQWLNRRANALLA
NGVELRDNQLVVPSEGLYLIYSQVLFKGQGCPSTHVLLTHTISRIAVSYQTKVNLLSAIKSPCQRETPEGAEAKPWYEP
IYLGGVFQLEKGDRLSAEINRPDYLDFAESGQVYFGIIAL

CASPON_mTNFα

Nucleotide sequence:
ATGCTGGAGGATCCGGAACGCAACAAAGAGCGAAAGGAAGCTGAGTTGCAAGCTCAAACCGCTGAGCAACA
CCATCATCACCATCATGGCAGCGGCGTGGATGTGGCGGATGTCCGTTCCAGCAGCCGTACGCCGAGCGATAAA
CCTGTCGCGCACGTAGTGGCGAATCCGCAAGCCGAGGGTCAGCTGCAGTGGCTGAATCGTCGCGCGAACGCG
CTGCTGGCCAATGGTGTTGAGCTGCGTGACAACCAACTGGTTGTTCCATCCGAAGGCCTGTACCTGATTTATTC
TCAAGTGCTGTTCAAAGGTCAGGGTTGCCCGAGCACGCACGTGTTGCTGACCCATACCATTAGCCGCATCGCA
GTCAGCTACCAGACCAAGGTCAACCTGTTGAGCGCGATCAAGTCCCCGTGTCAACGTGAAACGCCTGAGGGC
GCTGAGGCCAAGCCGTGGTATGAGCCGATCTACCTGGGTGGCGTGTTTCAGCTGGAGAAAGGTGACCGTCTG
AGCGCGGAAATCAACCGCCCGGATTATTTAGATTTTGCCGAGTCTGGTCAGGTGTACTTCGGCATTATTGCACT
G

Amino acid sequence:
MLEDPERNKERKEAELQAQTAEQHHHHHHGSGVDVADVRSSSRTPSDKPVAHVVANPQAEGQLQWLNRRANA
LLANGVELRDNQLVVPSEGLYLIYSQVLFKGQGCPSTHVLLTHTISRIAVSYQTKVNLLSAIKSPCQRETPEGAEAKPW
YEPIYLGGVFQLEKGDRLSAEINRPDYLDFAESGQVYFGIIAL

BIWA4 - single chain fragment variable

BIWA4

Nucleotide sequence:
ATGGAAATTGTGCTGACCCAGAGCCCGGCGACCCTGAGCCTGAGCCCGGGTGAACGTGCCACCCTGAGCTGT
AGCGCGAGCAGCAGCATTAACTATATCTATTGGTATCAGCAGAAACCGGGCCAGGCGCCGCGTCTGCTGATTT
ATCTGACCAGCAACCTGGCCAGCGGTGTTCCGGCGCGTTTTAGCGGCAGCGGTAGCGGCACCGATTTTACCCT
GACCATTAGCAGCCTGGAACCGGAAGATTTTGCGGTGTATTATTGCCTGCAGTGGAGCAGCAATCCGCTGACC
TTTGGCGGTGGCACCAAAGTGGAAATTAAACGTGGCGGCGGTGGCAGCGGTGGTGGTGGTAGCGGCGGTGG
CGGCAGCGAAGTGCAGCTGGTTGAAAGCGGTGGCGGCCTGGTGAAACCGGGTGGCAGCCTGCGTCTGAGCT
GTGCGGCGAGCGGCTTTACCTTTAGCAGCTATGATATGAGCTGGGTGCGTCAGGCGCCGGGCAAAGGCCTGG
AATGGGTGAGCACCATCAGCAGCGGCGGCAGCTATACCTATTATCTGGATAGCATCAAAGGCCGTTTTACCAT
TAGCCGTGATAACGCGAAAAACAGCCTGTATCTGCAGATGAACAGCCTGCGTGCGGAAGATACCGCGGTTTAT
TATTGCGCGCGTCAGGGCCTGGATTATTGGGGCCGTGGCACCCTGGTTACCGTGAGCAGC

Amino acid sequence:
MEIVLTQSPATLSLSPGERATLSCSASSSINYIYWYQQKPGQAPRLLIYLTSNLASGVPARFSGSGSGTDFTLTISSLEPE
DFAVYYCLQWSSNPLTFGGGTKVEIKRGGGGSGGGGSGGGGSEVQLVESGGGLVKPGGSLRLSCAASGFTFSSYD
MSWVRQAPGKGLEWVSTISSGGSYTYYLDSIKGRFTISRDNAKNSLYLQMNSLRAEDTAVYYCARQGLDYWGRGT
LVTVSS

CASPON_BIWA4

Nucleotide sequence:
ATGCTGGAGGATCCGGAACGCAACAAAGAGCGAAAGGAAGCTGAGTTGCAAGCTCAAACCGCTGAGCAACA
CCATCATCACCATCATGGCAGCGGCGTGGATGTGGCGGATGAAATTGTGCTGACCCAGAGCCCGGCGACCCT
GAGCCTGAGCCCGGGTGAACGTGCCACCCTGAGCTGTAGCGCGAGCAGCAGCATTAACTATATCTATTGGTAT
CAGCAGAAACCGGGCCAGGCGCCGCGTCTGCTGATTTATCTGACCAGCAACCTGGCCAGCGGTGTTCCGGCGC
GTTTTAGCGGCAGCGGTAGCGGCACCGATTTTACCCTGACCATTAGCAGCCTGGAACCGGAAGATTTTGCGGT

GTATTATTGCCTGCAGTGGAGCAGCAATCCGCTGACCTTTGGCGGTGGCACCAAAGTGGAAATTAAACGTGGC
GGCGGTGGCAGCGGTGGTGGTGGTAGCGGCGGTGGCGGCAGCGAAGTGCAGCTGGTTGAAAGCGGTGGCG
GCCTGGTGAAACCGGGTGGCAGCCTGCGTCTGAGCTGTGCGGCGAGCGGCTTTACCTTTAGCAGCTATGATAT
GAGCTGGGTGCGTCAGGCGCCGGGCAAAGGCCTGGAATGGGTGAGCACCATCAGCAGCGGCGGCAGCTATA
CCTATTATCTGGATAGCATCAAAGGCCGTTTTACCATTAGCCGTGATAACGCGAAAAACAGCCTGTATCTGCAG
ATGAACAGCCTGCGTGCGGAAGATACCGCGGTTTATTATTGCGCGCGTCAGGGCCTGGATTATTGGGGCCGTG
GCACCCTGGTTACCGTGAGCAGC

Amino acid sequence:
MLEDPERNKERKEAELQAQTAEQHHHHHHGSGVDVADEIVLTQSPATLSLSPGERATLSCSASSSINYIYWYQQKP
GQAPRLLIYLTSNLASGVPARFSGSGSGTDFTLTISSLEPEDFAVYYCLQWSSNPLTFGGGTKVEIKRGGGGSGGGGS
GGGGSEVQLVESGGGLVKPGGSLRLSCAASGFTFSSYDMSWVRQAPGKGLEWVSTISSGGSYTYYLDSIKGRFTISR
DNAKNSLYLQMNSLRAEDTAVYYCARQGLDYWGRGTLVTVSS

Recombinant human Growth Hormone

ompA_CASPON_rhGH

Nucleotide sequence:
ATGAAAAAGACAGCTATCGCGATTGCAGTGGCACTGGCTGGTTTCGCTACCGTAGCGCAGGCCCTGGAGGAT
CCGGAACGCAACAAAGAGCGAAAGGAAGCTGAGTTGCAAGCTCAAACCGCTGAGCAACACCATCACCATCAC
CATGGCAGCGGCGTGGATGTGGCGGATTTTCCGACCATTCCGCTGAGCCGTCTGTTTGATAATGCAATGCTGC
GTGCACATCGTCTGCATCAGCTGGCATTTGATACCTATCAAGAATTTGAAGAAGCGTATATCCCGAAAGAGCA
GAAATATAGCTTCCTGCAGAATCCGCAGACCAGCCTGTGTTTTAGCGAAAGCATTCCGACACCGAGCAATCGT
GAAGAAACCCAGCAGAAAAGCAATCTGGAACTGCTGCGTATTAGCCTGCTGCTGATTCAGAGCTGGCTGGAAC
CGGTGCAGTTTCTGCGTAGCGTTTTTGCAAATAGCCTGGTTTATGGTGCAAGCGATAGCAATGTTTATGATCTG
CTGAAAGATCTGGAAGAAGGTATTCAGACCCTGATGGGTCGTCTGGAAGATGGTTCACCGCGTACCGGTCAG
ATCTTTAAACAGACCTATAGCAAATTCGATACCAACAGCCATAATGATGATGCCCTGCTGAAAAACTATGGTCT
GCTGTATTGTTTCCGCAAAGATATGGATAAAGTGGAAACCTTTCTGCGCATTGTTCAGTGTCGTAGCGTTGAAG
GTAGCTGTGGTTTC

Amino acid sequence:
MKKTAIAIAVALAGFATVAQALEDPERNKERKEAELQAQTAEQHHHHHHGSGVDVADFPTIPLSRLFDNAMLRAH
RLHQLAFDTYQEFEEAYIPKEQKYSFLQNPQTSLCFSESIPTPSNREETQQKSNLELLRISLLLIQSWLEPVQFLRSVFAN
SLVYGASDSNVYDLLKDLEEGIQTLMGRLEDGSPRTGQIFKQTYSKFDTNSHNDDALLKNYGLLYCFRKDMDKVETF
LRIVQCRSVEGSCGF

Granulocyte – Colony Stimulating Factor

CASPON_G-CSF

Nucleotide sequence:
ATGCTGGAGGATCCGGAACGCAACAAAGAGCGAAAGGAAGCTGAGTTGCAAGCTCAAACCGCTGAGCAACA
CCATCACCATCACCATGGCAGCGGCGTGGATGTGGCGGATGCAACACCGCTGGGTCCTGCAAGCAGCCTGCC
GCAGAGCTTTCTGCTGAAATGTCTGGAACAGGTTCGTAAAATTCAAGGTGATGGCGCAGCACTGCAAGAAAAA
CTGGTTAGCGAATGTGCAACCTATAAACTGTGTCATCCGGAAGAACTGGTTCTGCTGGGTCATAGCCTGGGTA
TTCCGTGGGCACCGCTGAGTAGCTGTCCGAGCCAGGCACTGCAGCTGGCAGGTTGTCTGAGTCAGCTGCATAG
CGGTCTGTTTCTGTATCAGGGTCTGCTGCAGGCACTGGAAGGTATTAGTCCGGAACTGGGTCCGACACTGGAT

ACCCTGCAACTGGATGTTGCAGATTTTGCAACCACCATTTGGCAGCAGATGGAAGAATTAGGTATGGCACCAG
CGCTGCAGCCGACACAGGGTGCAATGCCTGCATTTGCAAGCGCATTTCAGCGTCGTGCCGGTGGTGTTCTGGT
TGCAAGCCATCTGCAGAGTTTTCTGGAAGTTAGCTATCGTGTTCTGCGTCATCTGGCACAGCCG

Amino acid sequence:
MLEDPERNKERKEAELQAQTAEQHHHHHHGSGVDVADATPLGPASSLPQSFLLKCLEQVRKIQGDGAALQEKLVS
ECATYKLCHPEELVLLGHSLGIPWAPLSSCPSQALQLAGCLSQLHSGLFLYQGLLQALEGISPELGPTLDTLQLDVADF
ATTIWQQMEELGMAPALQPTQGAMPAFASAFQRRAGGVLVASHLQSFLEVSYRVLRHLAQP

Parathyroid Hormone

CASPON_PTH

Nucleotide sequence:
ATGCTGGAGGATCCGGAACGCAACAAAGAGCGAAAGGAAGCTGAGTTGCAAGCTCAAACCGCTGAGCAACA
CCATCACCATCACCATGGCAGCGGCGTGGATGTGGCGGATAGCGTTAGCGAAATTCAGCTGATGCATAATCTG
GGCAAACATCTGAATAGCATGGAACGTGTTGAATGGCTGCGTAAAAAACTGCAGGATGTGCATAATTTTGTTG
CACTGGGTGCACCGCTGGCACCGCGTGATGCAGGTAGTCAGCGTCCTCGTAAAAAAGAAGATAACGTTCTGGT
TGAAAGCCACGAAAAAAGCCTGGGTGAAGCAGATAAAGCAGATGTTAATGTTCTGACCAAAGCCAAAAGCCA
G

Amino acid sequence:
MLEDPERNKERKEAELQAQTAEQHHHHHHGSGVDVADSVSEIQLMHNLGKHLNSMERVEWLRKKLQDVHNFV
ALGAPLAPRDAGSQRPRKKEDNVLVESHEKSLGEADKADVNVLTKAKSQ

SARS CoV 2 Nucleocapsidprotein

CASPON_NP

Nucleotide sequence:
ATGCTGGAGGATCCGGAACGCAACAAAGAGCGAAAGGAAGCTGAGTTGCAAGCTCAAACCGCTGAGCAACA
CCATCATCACCATCATGGCAGCGGCGTGGATGTGGCGGATATGTCTGATAATGGACCCCAAAATCAGCGAAAT
GCACCCCGCATTACGTTTGGTGGACCCTCAGATTCAACTGGCAGTAACCAGAATGGAGAACGCAGTGGGGCG
CGATCAAAACAACGTCGGCCCCAAGGTTTACCCAATAATACTGCGTCTTGGTTCACCGCTCTCACTCAACATGG
CAAGGAAGACCTTAAATTCCCTCGAGGACAAGGCGTTCCAATTAACACCAATAGCAGTCCAGATGACCAAATT
GGCTACTACCGAAGAGCTACCAGACGAATTCGTGGTGGTGACGGTAAAATGAAAGATCTCAGTCCAAGATGG
TATTTCTACTACCTAGGAACTGGGCCAGAAGCTGGACTTCCCTATGGTGCTAACAAAGACGGCATCATATGGG
TTGCAACTGAGGGAGCCTTGAATACACCAAAAGATCACATTGGCACCCGCAATCCTGCTAACAATGCTGCAATC
GTGCTACAACTTCCTCAAGGAACAACATTGCCAAAAGGCTTCTACGCAGAAGGGAGCAGAGGCGGCAGTCAA
GCCTCTTCTCGTTCCTCATCACGTAGTCGCAACAGTTCAAGAAATTCAACTCCAGGCAGCAGTAGGGGAACTTC
TCCTGCTAGAATGGCTGGCAATGGCGGTGATGCTGCTCTTGCTTTGCTGCTGCTTGACAGATTGAACCAGCTTG
AGAGCAAAATGTCTGGTAAAGGCCAACAACAACAAGGCCAAACTGTCACTAAGAAATCTGCTGCTGAGGCTTC
TAAGAAGCCTCGGCAAAAACGTACTGCCACTAAAGCATACAATGTAACACAAGCTTTCGGCAGACGTGGTCCA
GAACAAACCCAAGGAAATTTTGGGGACCAGGAACTAATCAGACAAGGAACTGATTACAAACATTGGCCGCAA
ATTGCACAATTTGCCCCCAGCGCTTCAGCGTTCTTCGGAATGTCGCGCATTGGCATGGAAGTCACACCTTCGGG
AACGTGGTTGACCTACACAGGTGCCATCAAATTGGATGACAAAGATCCAAATTTCAAAGATCAAGTCATTTTGC
TGAATAAGCATATTGACGCATACAAAACATTCCCACCAACAGAGCCTAAAAAGGACAAAAAGAAGAAGGCTG

ATGAAACTCAAGCCTTACCGCAGAGACAGAAGAAACAGCAAACTGTGACTCTTCTTCCTGCTGCAGATTTGGAT
GATTTCTCCAAACAATTGCAACAATCCATGAGCAGTGCTGACTCAACTCAGGCC

Amino acid sequence:
MLEDPERNKERKEAELQAQTAEQHHHHHHGSGVDVADMSDNGPQNQRNAPRITFGGPSDSTGSNQNGERSGA
RSKQRRPQGLPNNTASWFTALTQHGKEDLKFPRGQGVPINTNSSPDDQIGYYRRATRRIRGGDGKMKDLSPRWY
FYYLGTGPEAGLPYGANKDGIIWVATEGALNTPKDHIGTRNPANNAAIVLQLPQGTTLPKGFYAEGSRGGSQASSR
SSSRSRNSSRNSTPGSSRGTSPARMAGNGGDAALALLLLDRLNQLESKMSGKGQQQQGQTVTKKSAAEASKKPR
QKRTATKAYNVTQAFGRRGPEQTQGNFGDQELIRQGTDYKHWPQIAQFAPSASAFFGMSRIGMEVTPSGTWLTY
TGAIKLDDKDPNFKDQVILLNKHIDAYKTFPPTEPKKDKKKKADETQALPQRQKKQQTVTLLPAADLDDFSKQLQQS
MSSADSTQA

Interferon Gamma

CASPON_IFNγ

Nucleotide sequence:
ATGCTGGAGGATCCGGAACGCAACAAAGAGCGAAAGGAAGCTGAGTTGCAAGCTCAAACCGCTGAGCAACA
CCATCACCATCACCATGGCAGCGGCGTGGATGTGGCGGATCAGGATCCGTATGTTAAAGAAGCCGAGAACCT
GAAAAAATACTTTAACGCAGGTCATTCAGATGTGGCAGATAATGGCACCCTGTTTCTGGGTATTCTGAAAAACT
GGAAAGAAGAGTCCGATCGCAAAATCATGCAGAGCCAGATTGTTAGCTTCTACTTCAAACTGTTTAAAAACTTT
AAAGATGATCAGAGCATCCAGAAAAGCGTGGAAACCATTAAAGAAGATATGAACGTCAAATTCTTCAACAGCA
ACAAAAAAAAGCGCGACGATTTTGAGAAACTGACCAATTATAGCGTGACCGATCTGAATGTTCAGCGTAAAGC
AATTCATGAACTGATTCAGGTTATGGCAGAACTGAGTCCGGCAGCAAAAACCGGTAAACGTAAACGTAGCCAG
ATGCTGTTTCGTGGTCGTCGTGCAAGCCAG

Amino acid sequence:
MLEDPERNKERKEAELQAQTAEQHHHHHHGSGVDVADQDPYVKEAENLKKYFNAGHSDVADNGTLFLGILKNW
KEESDRKIMQSQIVSFYFKLFKNFKDDQSIQKSVETIKEDMNVKFFNSNKKKRDDFEKLTNYSVTDLNVQRKAIHELI
QVMAELSPAAKTGKRKRSQMLFRGRRASQ

Primer used for cloning of expression constructs

Table S2. Nucleotide sequences and functions of primers, nucleotides in lower case letters denote added sequences to facilitate cleavage with BsaI

Primer	Function	Sequence 5'-3'
1	T7AC to T7A3 forw	tgagACCGCTGAGCAACACCAT
2	T7AC to T7A3 rev	gcttcCAACTCAGCTTCCTTTCGC
3	Deletion 6His-GSG-VDVAD forw	GCCGCTGGTTCGATTACTA
4	Deletion 6His-GSG-VDVAD rev	TTGCTCAGCGGTTTGAGC
5	Deletion of GSG-VDVAD forw	ggtctcGCCGCTGGTTCGATTAC
6	Deletion of GSG-VDVAD rev	ggtctcagcggcATGATGGTGATGATGGTG
7	Deletion of VDVAD forw	CCGCTGGTTCGATTACTACC
8	Deletion of VDVAD rev	CGCCGCTGCCATGATGAT
9	Addition of SA-StrepII forw	ggtctcaAGCGCGTGGAGCCATCCGCAGTTTGAAAAAGGC AGCGGCGTGGATG
10	Addition of SA-StrepII rev	ggtctcgcgctATGATGGTGATGATGGTGTTGCTCAGC

Additional fermentation parameters

BIWA4

	Temp. [°C]	*CDM [g/L]*	*Vol. [L]*	*Duration [h]*	*μ [h^{-1}]*	*Generations*
Batch	37	6.25	9.00	7.25	-	-
Fed- Batch	30	44.11	15.54	25.00	0.1	3.61

Induction was carried out at feed hour 19 with an IPTG pulse added directly into the bioreactor corresponding to 0.1 mmol IPTG per litre of fermentation broth at the end of fermentation.

TNFα

	Temp. [°C]	*CDM [g/L]*	*Vol. [L]*	*Duration [h]*	*μ [h^{-1}]*	*Generations*
Batch	37	8.00	5.00	9.50	-	-
Fed- Batch	30	80.12	9.07	29.00	0.5	4.18

Induction was carried out at feed start with an IPTG pulse added directly into the feed medium corresponding to 0.5 µmol IPTG per gram of theoretical biomass at the end of fermentation.

Additional fermentation parameters

Fermentation parameters for rhGH, GCSF, PTH and IFNγ were identical as follows.

	Temp. [°C]	*CDM [g/L]*	*Vol. [mL]*	*Duration [h]*	*μ [h^{-1}]*	*Generations*
Batch	37	4.00	500	11.00	-	-
Fed- Batch	30	19.23	629	15.00	0.12	2.60
Fed- Batch	30	35.77	875	19.00	0.05	1.37

Induction was carried out at feed hour 19 with an IPTG pulse added directly into the bioreactor corresponding to 2 µmol IPTG per gram of biomass at the end of fermentation.

Specific and volumetric titers of all proteins produced

Table S3. Volumetric and specific titers of model proteins (total and stoichiometric titers corrected for the fusion tag)

	POI	Mass [kDa]	corr. factor	titer with tag [g/L]	titer corr. [g/L]	titer with tag [mg/g CDM]	titer corr. [mg/g CDM]
hFGF-2	construct 1	17091	-	4.8	4.8	75	75
	construct 2	18745	0.91	2.0	1.9	30	27
	construct 3	18045	0.95	0.9	0.8	14	13
	construct 4	21342	0.80	9.0	7.2	130	104
	construct 5	20639	0.83	9.5	7.9	140	116
	construct 6	22538	0.76	8.2	6.2	153	116
	construct 7	20639	0.83	10.8	9.0	160	132
	construct 8	19816	0.86	12.2	10.5	121	104
mTNF-α	native	17353	-	-	-	-	-
	non-T7-tagged	18876	0.92	1.1	1.0	28	26
	CASPON-tagged	21602	0.80	3.8	3.1	47	37
BIWA4	native	24876	-	1.6	1.6	44	44
	CASPON-tagged	29125	0.85	5.0	4.3	118	101
rhGH	native	22129	-	-	-	-	-
	CASPON-tagged	26379	0.84	5.2	4.4	197	165
G-CSF	native	18743	-	-	-	-	-
	CASPON-tagged	22992	0.82	2.3	1.9	82	67
PTH	native	9425	-	-	-	-	-
	CASPON-tagged	13674	0.69	2.2	1.5	65	45
IFNγ	native	16776	-	-	-	-	-
	CASPON-tagged	21026	0.80	1.9	1.5	58	46
NP	native	45626	-	-	-	-	-
	CASPON-tagged	49875	0.91	3.9	3.6	116	106

Reproducibility of lab scale fermentation

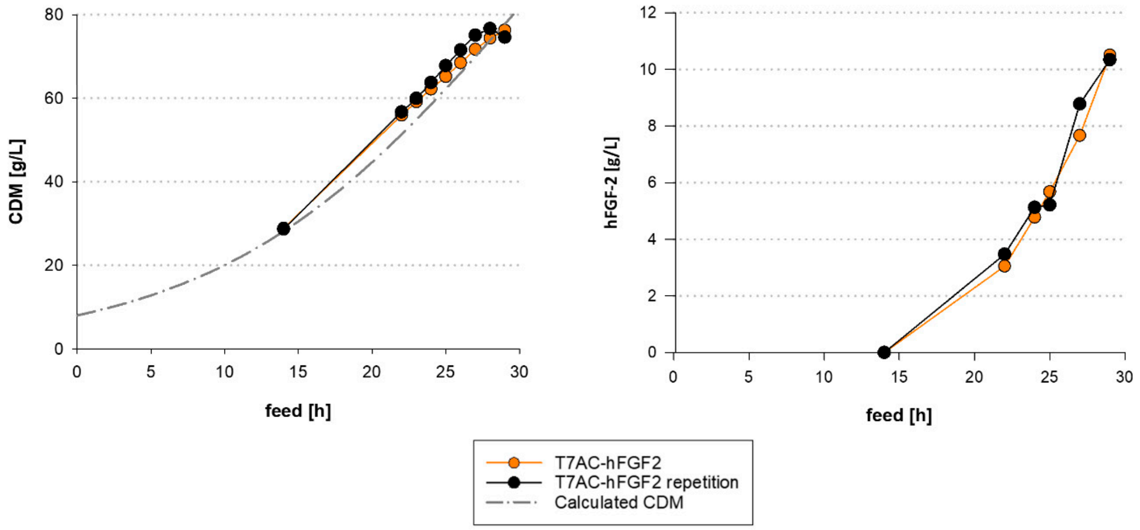

Figure S1
